# A Systemic Perspective on Organizations: International Experience with the Systemic Constellation Method

**DOI:** 10.1007/s11213-023-09642-2

**Published:** 2023-04-10

**Authors:** Salome Scholtens, Hedwig Boer, Lisa Kiltz, Joke Fleer

**Affiliations:** 1 Department of Health Sciences, Section Health Psychology, University of Groningen, University Medical Center Groningen, P.O. Box 1, Groningen, 9700 AD The Netherlands; 2 Faculty of Behavioural and Social Sciences, Department of Teacher Education, University of Groningen, Groningen, The Netherlands

**Keywords:** Systemic intervention, Systemic organizational constellation, Team coaching, Organizational transformation, Organizational coaching

## Abstract

A systemic perspective is considered helpful when facing complex organizational challenges, but its practical implementation may be challenging. A potential effective method that facilitates the practical application of a systemic perspective may be the Systemic Constellation method. This method aims to raise individuals’ awareness of their social context and to render explicit their tacit knowledge relating to this social context. In recent decades, consultants, coaches, and other professionals, worldwide, have adopted this method, acquired through self-education. However, thus far, this method received only little attention from the scientific community and scientific evidence on the effectiveness of the method is limited. There is currently almost no data on professionals using the Systemic Constellation method within organizations or on how and when they apply it. This lack of insights impedes its scientific evaluation and quality monitoring. We collected data from 273 professionals who use this method. Our results confirmed the existence of a diverse and growing international community. Respondents reported that the primary advantage of using this method is its perceived effectiveness. They felt that the method could benefit from a stronger scientific foundation. Our results shed light on a potentially effective and feasible method for applying a systemic perspective within organizations and suggest directions for future research.

**Supplementary Information:**

The online version contains supplementary material available at 10.1007/s11213-023-09642-2.

## Introduction

A systemic perspective applied to organizations and teams is considered beneficial when dealing with complex challenges, such as organizational change and transformation processes or persistent team dysfunction (Hawkins 2019; Hwang 2000; Maes and Van Hootegem 2019). According to Senge (1990), a systemic perspective enables the self-renewal of organizations and enhances their ability to adapt to changes in order to survive and thrive. Since the publication of Senge’s book, *The 5th Discipline*, in 1990, the systemic perspective has steadily gained popularity within the field of organizational consultancy and coaching. According to this perspective, organizations and teams can be viewed as social systems that function within a wider context. It recognizes dynamic interactions between the system’s individual components, which are in a state of constant flux, and enables clarification of the objectives, roles, values, and relations that characterize such systems (Bierema 2003; Harrington et al. 1999; Hwang 2000; Lawrence 2021). Within these social systems, the members’ knowledge about the system’s characteristics (i.e., its objectives, roles, and values) is assumed to rely on tacit knowledge (Andrews and Smits 2018; Barton et al. 2004), that is, implicit knowledge comprising habits, beliefs, values, and social structures relating to “how we do things here” (Lam 2000).

Whereas consultants, coaches, and leaders may be convinced of the importance of a systemic perspective on organizations and the usefulness of tacit knowledge, they may find it challenging to apply this perspective in their daily practice. The effective implementation of a systemic perspective within an organizational context necessitates its transformation into a practical approach or tool. Because tacit knowledge may only be present at the subconscious level, exclusive use of explicit and often verbal communication may not be sufficient. A well-known practical method that aims to access this tacit knowledge is Soft Systems Methodology (Checkland 2000).

Another method, thought to transform a systemic perspective into a practical approach and that enables access to tacit knowledge, is the Systemic Constellation method (Weinhold et al. 2014). This method aims to raise individuals’ awareness of their social context and to render explicit their tacit knowledge relating to this social context. It entails zooming out from the level of the individual to the wider level of the social context and encompasses a live visualization of the elements of a social system in a spatial way. The method is currently used in fields that include organizational change, leadership development, team coaching, and conflict resolution as well as for exploratory purposes, such as stakeholder analyses and strategy development (Burchardt 2015; Weinhold et al. 2014). In addition, the method is applied in the context of teaching and teacher development (Kopp and Martinuzzi 2013; Sipman et al. 2022). In the next section, we provide a more detailed description of the method and its practical implementation.

Globally, coaches, consultants, and other professionals seem to have embraced the Systemic Constellation method, leading to its expansion in recent decades. There are several training institutes across the globe as well as numerous books describing the practice. A literature review on the Systemic Constellation method (Scholtens et al. 2021) identified over 100 publications, mainly belonging to the category of popular literature. A recent Google search conducted in March 2023 on “Systemic Constellations training” resulted in over 1,000 hits. Moreover, an international organization has been established, currently with approximately 150 members, which offers certification for individual coaches and consultants as well as training institutes (see www.infosyon.com).

However, although the method seems to thrive, thus far, the method received only little attention from the scientific community and scientific evidence on the effectiveness of the method is limited (Scholtens et al. 2021; Weinhold et al. 2014). Consultants pursue self-education, drawing on the diverse landscape of courses, workshops, and books and generally apply it in various ways in combination with other methods in which they are proficient (Fasching 2009; Tenner 2014; Wakefield 2014).

Because of this learning-by-doing approach in which practice is combined with individual learning paths, there is currently almost no available data on professionals using the Systemic Constellation method within organizations or on how they apply it in their daily practice. This lack of insight relating to the community of practice and how and when the method is applied impedes scientific evaluation of, for instance, the method’s effectiveness as well as the establishment of quality standards for monitoring its use. Consequently, there is an entailed risk of ineffective and possibly harmful use (Groth 2004; Scholtens et al. 2021).

To gain insights into the community of practice and their needs is an important first step for advancing understanding of the method and its application. Hence, the aim of this study was to provide an overview of the field of international professionals who use the Systemic Constellation method within organizational settings and to advance understanding of why, when, and how the method is currently being applied. These insights will contribute to developing an approach for evaluating the method scientifically and push forward the development of evidence-based practice.

### The Systemic Constellation Method

The origins of the Systemic Constellation method lie in systemic family constellations applied in the fields of therapy and clinical counseling (Konkolÿ Thege et al. 2021). Commencing from 2000, systemic constellations have been further developed and applied to other social systems, such as organizations and teams (Scholtens et al. 2021; Weinhold et al. 2014). The method is based on a systemic-phenomenological perspective (Sipman et al. 2022). A premise of the method is that individuals hold intuitive knowledge about the structures, relations, and interdependencies of the components within the social systems of which they are part. However, this knowledge is often implicit and subconscious, although it can be rendered explicit.

To this end, systemic constellations are implemented to visualize a social system using a spatial arrangement of elements relevant to the social context. These elements mainly comprise individuals or objects representing functions or roles within the social system (e.g., manager), groups, or stakeholders (e.g., customers or patients). They may also include concepts or societal aspects (e.g., a value or education). Accordingly, the social system and its interrelated components can be rendered visible and tangible. The method is often applied in a workshop setting comprising 10–30 participants and a trained facilitator. Often, one of the participants introduces a case, which is then addressed collectively by the group during a session. Elements relevant to the case and its social context are defined and placed on a table or somewhere in the room. Subsequently, the facilitator guides the participant’s and group’s exploration of the social system’s structures, interdependencies among its elements, and different perspectives. This guided process, during which the facilitator stimulates individual and shared reflections, allows individuals to explore perspectives other than their own relating to organizations, teams, or any other social context. Besides its application in groups, the method can also be applied in individual coaching contexts. A detailed description of its application is described in a previous publication (Scholtens et al. 2021) and in the online Supplementary Material.

## Method

A digital questionnaire that included both open-ended and closed-ended questions was designed using Qualtrics software (USA). It was administered to members of the international community of consultants, coaches, and trainers, via the networks of Infosyon, large training institutes, such as the Bert Hellinger Institute in the Netherlands (www.hellingerinstituut.nl), and the Wieslocher Institute for Systemic Solutions in Germany (www.wieslocher-institut.com) as well as institutions within our own networks. Moreover, we encouraged respondents to distribute the questionnaire within their own networks. This snowball sampling method served our purpose of reaching a wider international community. To ensure international representation, the survey was available in five languages: Dutch, English, German, Portuguese, and Spanish. We chose these languages because they are used in existing publications on the Systemic Constellation method (Scholtens et al. 2021) and were commonly encountered by Infosyon and by the training institutes that we consulted. We therefore expected that they would be the languages most widely used by professionals applying the Systemic Constellation method.

The questionnaire covered sociodemographic items including gender, age, nationality, and employment status. Furthermore, the respondents were asked for how long they had used the method, in which settings, and for what issues. Respondents were also asked how they were trained in its use and whether they combined it with other methods. Additionally, we included open-ended questions to gather more details about the trainers’ motivations and experiences relating to the Systemic Constellation method. We asked the respondents what sparked their interest in the method, what advantages and disadvantages they perceived in using the method, and whether they had any experience of applying it in a counterproductive way. Finally, respondents reported on what they felt could help them as users of the method to enhance the quality of their work and what was needed within the broader community to improve the quality of the method more generally.

The survey was piloted with eight respondents (professionals belonging to the target population, who had over 10 years of experience of using the method) from different language areas. We adjusted the items according to the feedback we received from the pilot participants before administering the questionnaire to coaches, consultants, and trainers within the international community, who use the Systemic Constellation method in organizational settings. Respondents received an invitation to participate, which included information about the study and contained a link to the questionnaire. To be eligible to participate in the study, respondents were required to be above 18 years of age and to have prior experience in applying the Systemic Constellation method within an organizational setting or be planning to do so. Individuals who neither had experience in applying this method nor were planning to do so were excluded from the study. Besides these criteria, no further exclusion criteria were set because we had no prior knowledge regarding the experience levels of the professionals. We included participants who were planning to apply the Systemic Constellation method because these respondents could represent young professionals or those embarking on their careers, who had limited experience. The complete questionnaire and the letter providing information on the study are included in the online Supplementary Material.

We performed an inductive content analysis on the qualitative data extracted from the open-ended questions. Two researchers independently coded the data using the codebook developed after the initial round of coding. In cases of disagreement over coding, a third researcher was included in the discussion, and all three researchers shared their rationales for the coding until a consensus was reached. Answers that could not be interpreted by any of the authors were not coded. The quantitative data were analyzed using IBM’s SPSS Statistics program, version 23.0. We mainly performed descriptive analyses.

The study was approved by the ethics committee of the research institute (approval number: 202,000,571) and was pre-registered in the Open Science Framework (https://osf.io/4buzw) as part of a larger study being conducted on the Systemic Constellation method. All of the respondents provided their informed consent. To ensure privacy and confidentiality, the survey was completed anonymously and no personal data were collected. If respondents wished to receive information about the results of the study, they could provide their email addresses at the end of the survey. These email addresses were stored separately from the survey data to retain anonymity.

## Results

### Respondents’ Characteristics

**Fig. 1:**
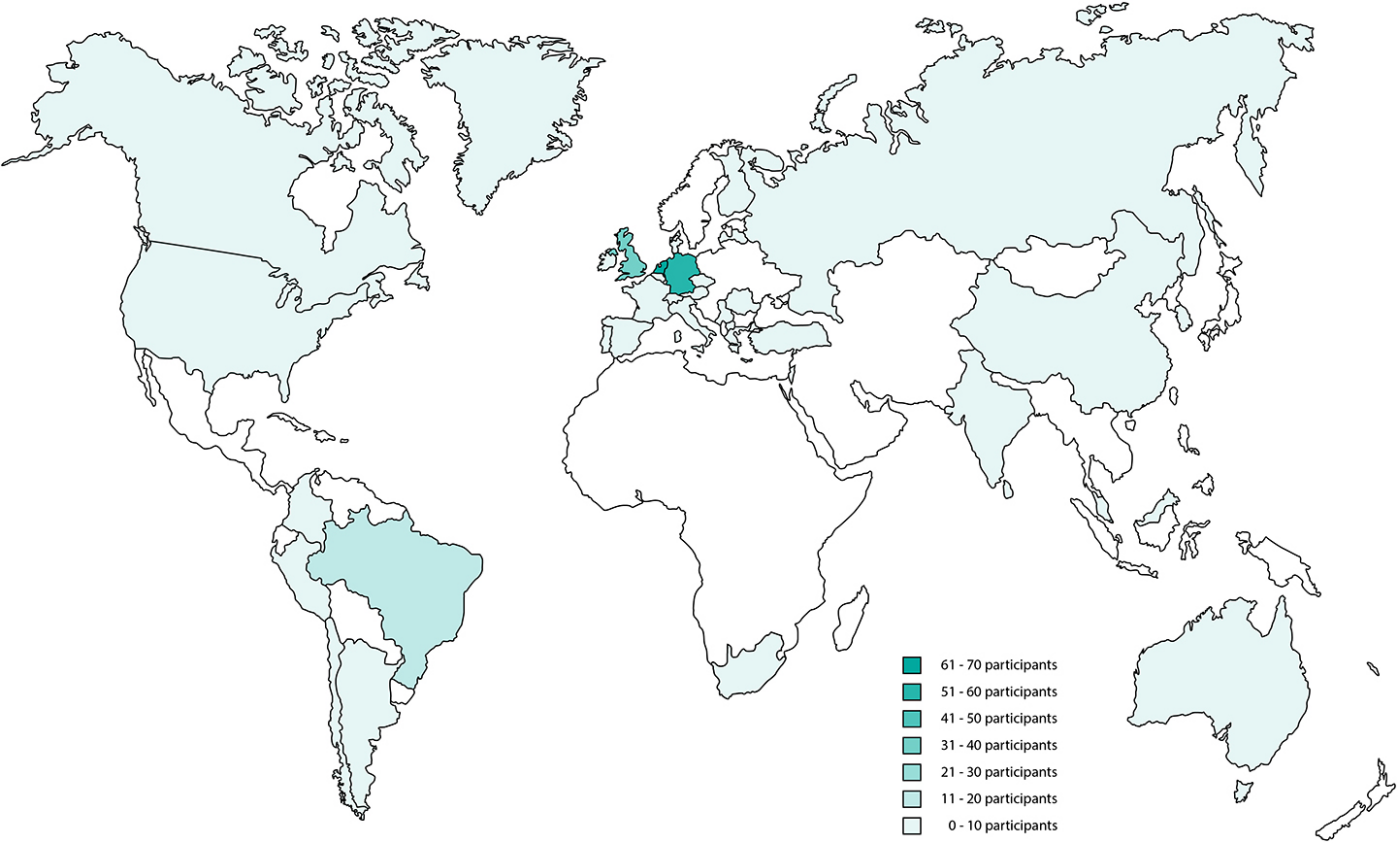
Global distribution of locations where the respondents applied the Systemic Constellation method within an organizational setting

Between December 14, 2020 and June 22, 2021, 319 professionals participated in the survey. Those who indicated that they never used the method and were not planning to do so were not included in the study (n = 46). In total, 273 respondents used (n = 260) or planned to use (n = 13) the method. Six of these respondents indicated that they no longer used the method, for instance, because they had changed their job or had mainly used another method. The majority of the respondents were female (n = 173; 63%). More than half of the respondents were 41–55 years old (n = 158; 58%), another 7% (n = 19) were 26–40 years old, 32% (n = 86) were 56–70 years old, and 2% (n = 6) were aged above 70 years. Table 1 presents an overview of the respondents’ characteristics. Most respondents were freelancers (self-employed and without personnel) and most worked as a consultant or coach. In general, respondents had 4.4 years of (often part-time) education in the systemic organizational constellation method. Most respondents originated from Germany, the Netherlands, or the UK, but the study showed almost global coverage. Figure 1 shows the international distribution of the participants.

**Table 1:** Descriptive characteristics of the professionals using the Systemic Constellation method within organizational settings and their application of the method*

Item	Response categories	N (%)
Current working situation	Freelancer/self-employed	157 (58)
	Self-employed/business owner: with personnel	41 (15)
	Employed: fixed contract	52 (19)
	Employed: temporary contract	6 (2)
	Retired or unemployed	10 (4)
	Other	3 (1)
Role or function	Coach	64 (23)
	Consultant/adviser	58 (21)
	Facilitator	46 (17)
	Constellator (i.e. a persons who performs systemic constellations)	35 (13)
	Trainer	17 (6)
	Therapist	15 (6)
	(Project) manager, CEO, business owner	10 (4)
	HR manager/specialist	5 (2)
	Teacher	5 (2)
	Researcher	4 (2)
	Other	6 (2)
Starting period of use of the method	1990–1995	7 (3)
	1996–2000	17 (6)
	2001–2005	29 (11)
	2006–2010	45 (17)
	2011–2015	54 (20)
	After 2015	106 (39)
Frequency of use	One or more times a week	87 (32)
	One or more times a month but less than once a week	95 (35)
	One or more times a year but less than once a month	63 (23)
Frequency of performing a constellation with people as representing elements, when using the method	Never	6 (2)
	Rarely	38 (14)
	Sometimes	63 (23)
	Regularly	58 (21)
	Often	46 (17)
	Always	21 (8)
Frequency of combining the method with other methods	Never	10 (4)
	Rarely	23 (8)
	Sometimes	55 (20)
	Regularly	67 (25)
	Often	54 (20)
	Always	23 (8)
Settings in which the method is applied**	Individual coaching	190 (70)
	Team coaching	150 (55)
	Organizational coaching or consultancy	167 (61)
	Education	69 (25)
	Research	35 (13)
	Therapy	44 (16)
	Other	28 (10)

Notes: *Some percentage may not add up to 100% due to missing values; ** multiple answers were possible

### Application of the Method

**Fig. 2:**
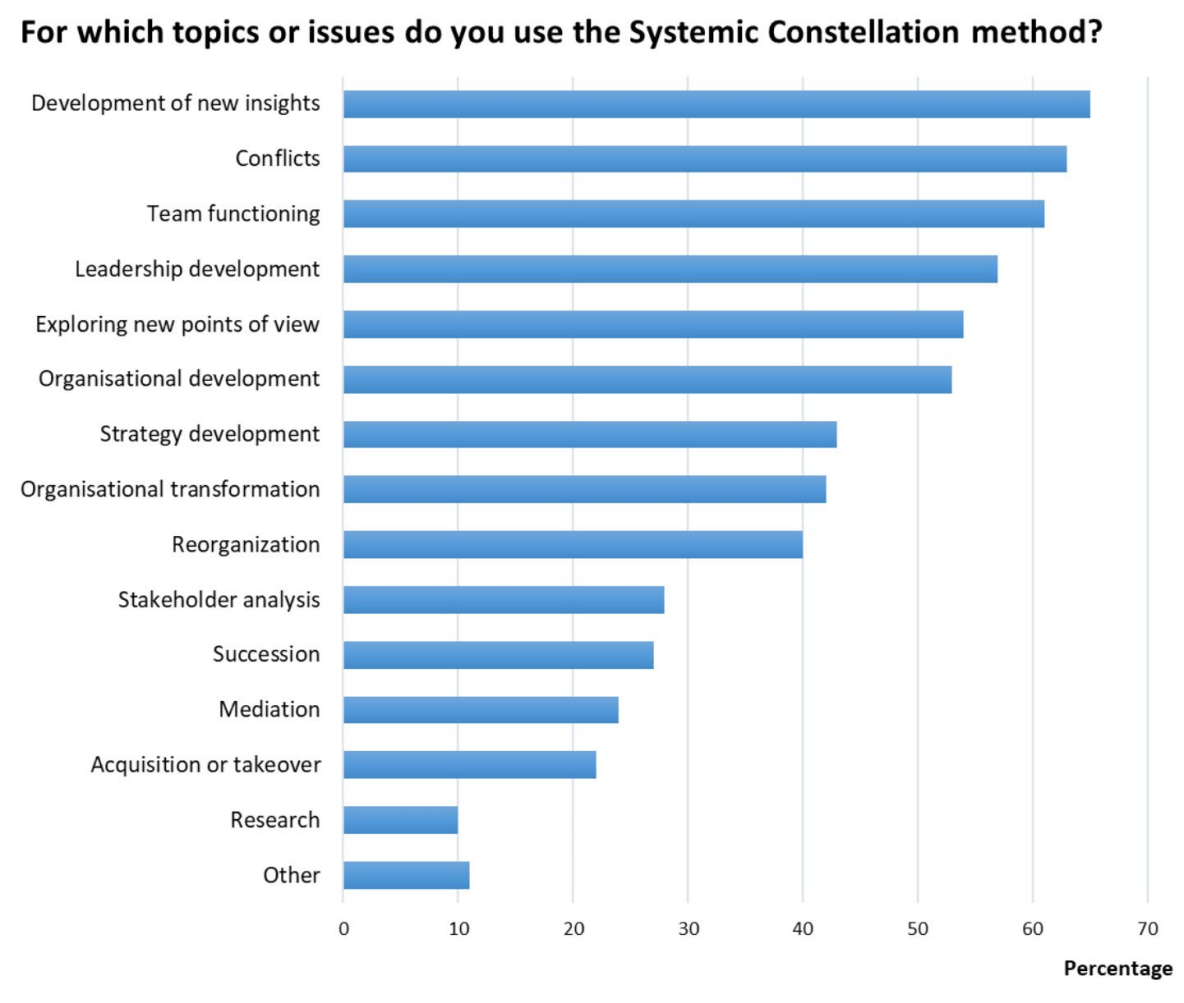
Frequency of use of the Systemic Constellation method for specific topics or issues. The bars show the percentages of respondents who reported using the method for that topic

**Fig. 3:**
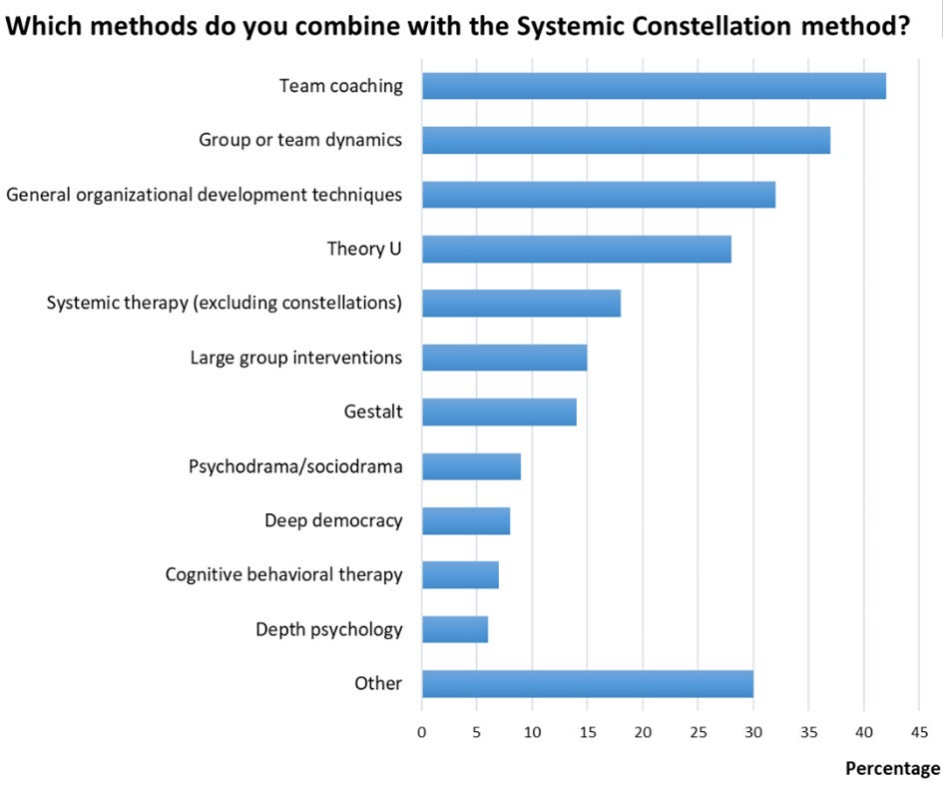
Frequency of use of the Systemic Constellation method with other specified methods or techniques. The bars show the percentages of respondents who reported using a specific method or technique in combination with the Systemic Constellation method. Methods and techniques reported under the “Other” response category included general coaching techniques, neurolinguistic programming (NLP), spiral dynamics, and mindfulness methods

The results of the study showed that the respondents applied the method regularly, using it in individual, team, and organizational coaching settings and for a wide range of topics (see Table 1; Fig. 2). More than 60% of respondents indicated that they used the method for the development of new insights, conflict resolution, and team functioning. Additionally, more than 50% of respondents reported using it for organizational development, exploring new points of view, and leadership development. Apart from the above pre-defined options, respondents mentioned the following areas of application: marketing, brand development, career development, and personal development. 60% of the respondents (n = 163) also applied the method in an online setting. Moreover, the respondents applied the method in small, large, and multinational companies or institutes (with numbers of employees ranging from 1 to over 50,000) and in a wide variety of industries and sectors (for a complete list, see the online Supplementary Material). Half of the respondents (50%; n = 136) believed that they needed to be familiar, at least to some extent, with the intrinsic laws and values of a company or industry to apply the method effectively, while 28% (n = 77) felt that this was not necessary. Half of the respondents combined the method regularly with other methods (53%; n = 144). The most commonly mentioned methods that were combined with constellations were those focusing on group dynamics, team coaching techniques, general organizational development techniques, and Theory U (see Fig. 3). In addition to the pre-defined options, respondents reported using general coaching techniques, neurolinguistic programming (NLP), spiral dynamics, and mindfulness methods.

### Positive Experiences with the Method

Most often, respondents referred in the open question to specific characteristics of the method that they considered advantageous, such as its perceived effectiveness (37%) and the clarity it could provide on understanding of hidden dynamics (15%) (Table 2). In addition, the depth of the method (11%), an emphasis on embodied experience (14%), the ability to visualize systems (9%), and the use of a systemic perspective (6%) were mentioned as advantages associated with the method. Beneficial aspects pertaining to coaches or consultants mentioned by the respondents included their own development (20%) or that of their profession (16%). Some respondents (10%) perceived the method as an additional coaching tool and a valuable addition that enriched their toolbox. Table 2 illustrates all of the codes used for the open-ended question, “What are the personal advantages that you experience from using the method?”, with examples drawn from the data, and numbers of respondents whose responses were categorized under the relevant codes.

**Table 2:** Responses to the open-ended question, “What are the personal advantages that you experience from using the method?”

Code		Description	Examples of responses	N (%)*
***Advantages related to characteristics of the method***				
	**Perceived effectiveness**	Respondents considered the method’s perceived effect and the insights acquired from using the method as advantages. They mentioned a long-lasting effect, a solution-focused orientation, help with decision making, and the ability of the method to loosen a system or let it ‘flow’ again.	*“The effect works deeply and has lasting results.”* *“Insights about projects, relationships, proposals and many other areas that can be helpful in decision making.”*	102 (37)
	**Clarity and reduction of complexity**	An advantage of the method described by respondents is the bigger picture it provides on an issue as well as the clarity it provides on understanding of hidden dynamics in a social system or reduction of complexity.	*“Revealing the hidden dynamics, helping people see the bigger picture.”* *“It gives me clarity about the hidden dynamics at work in the entrepreneurial system.”*	41 (15)
	**Embodiment**	Respondents mentioned embodiment or the use of sensations instead of cognition and verbal expressions as an advantage offered by the method. It provides a “different language,” by allowing for communication without words.	*“Getting a language for what I observe when I work with groups.”* *“Solutions work in a non-cognitive way.”*	39 (14)
	**Depth**	Respondents described the depth, richness, or wholeness that the method provides as advantages. They mentioned that the method touches on the heart of an issue.	*“Working at a deeper level with coaching clients.”* *“Get to the heart of the issue quickly.”*	30 (11)
	**Visualization**	Respondents described the visualization of a social system or a situation as an advantage offered by the method. They valued that the method helps to make visible unknown or hidden dynamics.	*“Present projects, goals, teams, conflicts, ideas, thoughts and many other topics/situations and make their dynamics visible.”* *“Make systems visible.”*	25 (9)
	**Systemic perspective**	Respondents specifically mentioned the systemic perspective or the alternative view the method provided as an advantage.	*“Systemic insight in a very short time.”* *“Using a systemic approach to diagnostic processes with teams.”*	17 (6)
	**Client centered**	Respondents appreciated the method’s client-centered focus in which clients help to shape the solution, and the entire method centers on the participation of clients.	*“To be able to help others substantially with their issues.” “To help other people.”* *“The client works and we follow.”*	17 (6)
	**Healing**	Respondents mentioned that the method has a “healing” effect, which could be an advantage.	*“Each constellation [is] a healing process.”*	8 (3)
	**Without judgment**	Respondents felt that this method reveals the current situation and is free of judgment, which they viewed as an advantage.	*“Look without judgment.”*	4 (1)
***Advantages related to coaches or consultants***				
	**Own development**	Respondents described how the method enabled them to develop at a personal or professional level. They also stated that it made sense and provided life satisfaction.	*“I got knowledge about myself, which makes me a better leader.”* *“[I developed] greater self and social awareness and more self-efficacy in my social and professional life.”*	55 (20)
	**Professional benefits**	Respondents mentioned work-related benefits, such as belonging to a professional community or financial advantages.	*“I attain results quicker and make a difference in my job.” * *“Money-making activity doing what I love to do.”* *“Being part of a like-minded community.”*	45 (16)
	**Autonomy and own role**	Respondents considered a coach’s experience of autonomy and how they perceive their own role within this process as advantages.	*“Interesting combination of the therapist’s role with regard to the client’s process.”* *“My own effectiveness and a deeper and more honest cooperation than are usually possible in a business context.”*	29 (11)
	**Enriching toolbox**	Respondents stated that the method was a valuable addition to their existing repertory that was relevant to their work. Some stated that it provided a potential opportunity for addressing problems or questions in their working life. It was described as an enriching tool or method for them to use and they valued the innovative nature of the method.	*“[It is] an innovative method that allows diagnosis and testing of solutions.”* *“A new tool to offer clients.”*	28 (10)
	**Joy**	Respondents appreciated the joy, fun, and sense of gratification that they experienced when using the method.	*“Gratification”* *“Fulfillment.”* *“That it brings ease and simplicity and joy.”*	12 (4)

Notes: *Out of 273 respondents 213 (78%) responded to this question. Respondents could provide multiple responses, which may have resulted in multiple codes per respondent. A total of five responses were unclear and could not be coded

In connection with the advantages of using the method, respondents most often reported its perceived effectiveness as the reason for their initial interest in it (22%) (Table S1, online Supplementary Materials). Other characteristics of the method, such as its depth (10%), an emphasis on embodied experience (10%), the ability to visualize systems (9%), and the use of a systemic perspective (8%) were listed as reasons that sparked the respondents’ interest. In addition, some saw an opportunity for developing their own profession (8%) and acquiring an additional coaching tool (5%). However, some respondents did not specify what aspects sparked their interest; instead, they mentioned how they got interested in the method, for instance, because of their own personal or professional experiences (36%) or because it was recommended by someone else (9%).

### Disadvantages and Counterproductive Effects

The perceived disadvantages of the method mentioned by the respondents in the open question could be distinguished as those related to the method’s characteristics, to the clients or the area of application, and to the coach or consultant (Table 3). The respondents mentioned the following difficulties associated with the method: difficulty explaining the method (9%), possible unpleasant outcomes (6%), practical issues (4%), lack of certification (4%), a weak scientific basis (3%), and a large interpretative leeway (3%). An important disadvantage relating to clients was their mistrust or “reluctance” to try the method (15%). Moreover, respondents mentioned dependence on client requirements (e.g., their willingness to participate), as well as the restricted context of the method’s application (8%), the early stage of the market (5%), and financial matters (5%). Thirty-nine respondents (14%) did not perceive any disadvantages. Table 3 shows all of the codes that emerged from the open-ended question: “What are the personal disadvantages that you experience from using the method?”, with examples extracted from the data, and numbers of respondents whose responses matched with the relevant codes.

**Table 3:** Responses to the open-ended question, “What are the personal disadvantages that you experience from using the method?”

Code		Description	Examples of responses	N (%)*
***Disadvantages related to characteristics of the method***				
	**Difficult to explain**	Respondents referred to a lack of understanding in general about the method (beyond just the client) as a disadvantage. The method could be difficult to explain.	*“The fact that I cannot communicate easily about what it is that I do with the method [is a disadvantage].”* *“People do not understand it, and it is still difficult to explain. It seems better not to explain and to just let people experience it, but this is not easy either.”*	24 (9)
	**Risky**	Respondents noted that that the method could result in an unpleasant outcome or that some situations could be (too) sensitive for its use, perhaps because it exposes problems that the organization is not ready to discuss.	*“I experience it as a fantastic way of working, but also as a risky one.”* *“It quickly goes very deep emotionally, so it is a task entailing a lot of responsibility for me to design the process constructively, for example, to avoid re-traumatization.”*	16 (6)
	**No certification**	Respondents felt that the increased number of individuals using this method is resulting in a reduction in quality. They also mentioned that no quality checks exist relating to the use of this method.	*“Systemic working has become more widely known in recent years; likewise, the numbers of people who present themselves as a systemic therapist/coach/adviser [has increased]. This [situation] clouds the market. The supply is increasing, but the average quality is decreasing.”* *“The method and the trainings should be offered at the university level*”	10 (4)
	**Practical matters**	Respondents referred to the practicalities in implementing the method, such as COVID-19 measures and the requirements of a constellation, such as space, as disadvantages.	*“Full constellations are difficult for organizations to arrange in terms of space and representatives.”* *“You need to ‘rent a crowd’ for organizational constellations if you want to use people.”*	10 (4)
	**Interpretative leeway**	Respondents expressed the view that the method is too dependent on the interpretations of the people involved, namely the coach as well as the clients’ interpretations.	*“Misinterpretation of what appears to be unfolding [is a disadvantage]. I find that tricky, especially within organizational constellations.‘”* *“Some participants make more of it than I see in it. So sometimes things take on a life of their own.”*	9 (3)
	**Weak scientific basis**	Respondents lacked solid ground for this method as the theoretical background or scientific evidence is limited.	*“[It is] not possible to explain origins or why it works.”* *“[There is a] lack of facts supporting the method and its approach in practice.”*	9 (3)
	**Esoteric**	Respondents observed that the method is considered too “woolly” or unprofessional or it is considered to have a mystique or be esoteric.	*“That sounds spooky for some people.”* *“It is taken as esoteric practice”*	8 (3)
***Disadvantages related to clients or areas of application***				
	**Reluctance**	Respondents indicated that clients’ mistrust toward the method is a disadvantage. Also, they have mentioned little credibility, skepticism of clients as disadvantages.	*“Companies in many countries are not very open to the method, so it is hard to find clients and work with the method systematically.”* *“Some people or associates may have been judgmental or afraid when I talked about this work.”* *“High perceived weirdness index.”*	42 (15)
	**Client and context requirements**	The dependence of the method’s effectiveness on the client’s willingness to use it and whether or not the organization is open to its use can be disadvantages. There may be a limited scope or application area for applying the method. Moreover, respondents noted that some groups are not ready for it or refer to what a client can handle when a certain required attitude is lacking.	*“It quickly shows hidden dynamics that sometimes the client doesn’t want to deal with”* *“The method could only be used to a limited extent in my previous area of work.”*	21 (8)
	**Early stage of the market**	Respondents believed that the market for this method is still quite nascent and unknown and that people are not yet familiar with the method.	*“I have never attempted to earn a living from organizational constellations because it still feels like an edgy space for many people—not widely understood or accepted—and so the market is still young, I would say.”* *“There is still little demand for organizational constellations.”*	15 (5)
***Disadvantages related to coaches or consultants***				
	**Financial matters**	Respondents referred to the financial disadvantages of using this method, such as insurance, education costs, and low profitability.	*“I have done a lot of training that took time and money but have not yet used it directly to generate any income.”* *“[It is] not yet widely accessible via health insurance.”*	14 (5)
	**Impact on the coach**	Respondents considered the efforts that the coach has to make as a disadvantage.	*“It also requires something from the trainer. After a constellation, I also need some space to let it go, and that takes time and time is money.”* *“Constellations are exhausting even for trainers.”*	6 (2)
**None**		Respondents explicitly stated that there are no disadvantages associated with the method.	*“I experience no disadvantages.”*	39 (14)

Note: *Out of 273 respondents, 180 (66%) responded to the question. Respondents could provide multiple responses, which may have resulted in multiple codes per respondent. A total of 11 answers were unclear and could not be coded

A minority of the respondents (n = 37) reported that they experienced counterproductive effects. Examples included the experience of the method being overly confrontational or confusing for clients (n = 9), touching on previous traumas (n = 4), and clients’ frustration (n = 3). Eight respondents reported that they encountered too much resistance to be able to work effectively with the method, and five mentioned that in general, application of the method was unsuccessful. Some respondents (n = 22) speculated on possible reasons for these counterproductive effects. They believed that the method was not applied correctly or that the facilitator should have introduced the method in a more effective way and provided a stronger grounding context and a safer environment. Additionally, they thought that the facilitator was not sufficiently trained, did not have enough experience, or was too dominant to achieve an effective constellation.

### Needs Assessment

Responses to the open-ended questions, “What would be, for you as a user of the method, most helpful to improve the quality of your work?” and “What does the field (i.e., the community of practitioners) of the systemic organizational constellation method need?” revealed various needs related to the method, to the clients or the field, and to the coach or consultant. Those related to the method itself were the requirement for more (scientific) research on the method (7%), availability of a good description or a guidebook (7%), and certification of coaches and training institutes (3%) (Table S2 in the online Supplementary Materials). Respondents mentioned the following needs relating to the clients and field of application: more acceptance among clients (7%) and more possibilities for applying the method (3%). Finally, needs relating to the coaches themselves, mentioned by respondents, were more practice with the method (12%), more peer-to-peer support (12%), continuous learning and self-development (11%), and being connected to a community (7%).

Respondents mentioned the following needs for the field as a whole: a solid scientific foundation (10%), increased availability of affordable materials, and high-quality training programs (7%) (Table S3, online Supplementary Materials). Moreover, they stated that demystification of the method (3%), so that the method would not evoke esoteric associations, would be beneficial. Furthermore, the method should be more extensively marketed (10%) and should gain more recognition (8%). In addition, they mentioned the need to improve the quality of application of the method, possibly through the introduction of certification for coaches and consultants (9%). Within the field, respondents pointed to the need for more unity, less competition, and more openness to other methods (7%) as well as an (international) community (4%).

## Discussion

To the best of our knowledge, this is the first study to provide an overall picture of the professionals who apply a systemic perspective using the Systemic Constellation method within an organizational setting. Our findings reveal that professional users of this method constitute a broad, international community and that the number of professionals trained in the method has steadily increased over the years. Moreover, the method is applied across a range of coaching or consultancy settings and within diverse sectors and industries.

Our findings further illuminated the initial motivations of users, perceived benefits as well as disadvantages of using the method, and the wider needs of the professional community. The perception of the method’s effectiveness was one of the main aspects that initially sparked professionals’ interest, in addition to being the main advantage ascribed to the method. Although the method’s actual effectiveness was not scientifically verified in this study, this perception is in line with the findings of previous studies (Boland and Michaelis 2006; Gutmark 2014; Kolodej et al. 2016). The specific characteristics of the method that were highly valued included the use of a systemic perspective, the emphasis on an embodied experience, and the possibility of visualizing the system. Furthermore, respondents mentioned that the method facilitated an understanding of hidden dynamics within a team or organization and enhancing the individual’s ability to see the bigger picture. These observations endorse the value of this method in applying a systemic perspective practically to teams or organizations.

The visualization aspect, which was specifically mentioned by the respondents, has also been reported by Kopp and Martinuzzi (2013) as a benefit associated with the Systemic Constellation method. Specifically, they argued that visualization enables an issue and its context to be captured in one glance. Visualization is also a feature of other methods applied within organizational settings, such as rich picture building within the Soft Systems Methodology, wherein drawing constitutes the first step in the exploration of a problem and the generation of insights on the system (Checkland 2000). Like the Systemic Constellation method, drama-based methods, for example, psychodrama and socio-drama, entail visual spatial representation of relationships and externalization of the client’s internal picture with the aid of role playing (Schumacher 2000). A key difference between these approaches is that while psychodrama focuses on behavior of an individual or a group, the Systemic Constellation method in addition aims to bring the underlying tacit knowledge of the individuals on the social context to the surface. Moreover, the Systemic Constellation method has a stronger emphasis on a systemic perspective for addressing an issue (Schumacher 2000).

The method’s disadvantages, as identified by the professionals, were a weak scientific basis, the absence of certification and quality monitoring, interpretative leeway, and the appearance of being esoteric. These concerns match those expressed in literature (Groth 2004; Scholtens et al. 2021). The respondents attributed potentially unpleasant outcomes and counterproductive experiences to suboptimal application or insufficient training, emphasizing a pressing need for greater professionalization of the field and scientific evaluation of the method. Development of a scientific foundation and of measures for quality assurance as well as high-quality educational materials and training programs were identified as priority needs during the needs assessment. Although Infosyon offers certification of training institutes and individuals, this fact is either not widely known within the international community or it may not be the type or level of certification that the respondents desire.

Overall, a priority emerging from the needs assessment was improvement of the quality of the method itself, its application, and professional users. To this end, greater (scientific) understanding of the method is needed along with evidence of its effectiveness and measures to assure and monitor its quality. To improve the quality of the coaches, respondents pointed to the need for more opportunities for self-education and training and access to high-quality educational materials, training programs, peer-to-peer networks, and supervision. Over time, improved quality may lead to more professional application, which could serve to counteract the reluctance and lack of understanding on the part of clients, as observed by the respondents. Moreover, quality assurance could lead to greater publicity and acceptance of the method at a professional level. Other methods used in the coaching and consultancy field have followed similar paths, often emerging from practice (Grant and O’Connor 2019). One example is mindfulness-based cognitive therapy (Segal et al. 2013). Although meditation is an age-old practice, the creation of a standardized protocol and training on meditation practice facilitated the assessment of the method’s purposefulness and effectiveness in research. As a result, a standardized approach can be used to teach the method to others. These developments prompted widespread acceptance of mindfulness-based cognitive therapy, which has been thoroughly researched and has evolved into an evidence-based intervention that is now covered by many health insurance companies.

A strength of the present study is its large scale and the broad, international distribution of the survey respondents, who were professional users of the Systemic Organizational constellation method. Furthermore, the open-ended questions allowed for an exploration of the professionals’ perspectives and opinions. The qualitative data was coded by two independent researchers, with the involvement of a third researcher in case of disagreement, thereby increasing the reliability of the data extraction process. Nevertheless, we acknowledge the following limitations. Despite the many open-ended questions included in the survey, the amount of information that respondents could provide was limited. Moreover, some of the questions could have been misunderstood or misinterpreted. To counter such misunderstandings, we performed a pilot survey. Another limitation concerned the respondents’ nationalities, as the majority were located in Western Europe. The reasons for this uneven distribution of nationalities may be that in general, more professionals use the method in that region. Given that the actual number of professionals using the method is unknown, we cannot draw any definitive conclusions regarding the proportion of professionals included in the study or whether the distribution of professionals, languages, and countries approximated the reality. Despite this limitation, the large number of respondents relative to Infosyon members, numbering around 150 professionals, combined with the study’s global coverage makes us confident that the study reached a considerable proportion of the actual professionals who are currently using the method. Furthermore, the study may have mainly included professionals who are satisfied with the method. Although this limitation will probably have a small effect on the results describing the community and their needs, less weight should be given to the reported perceived effectiveness.

The results of this study conducted among the international community of professionals reveal that the Systemic Constellation method is potentially an effective and feasible approach for applying a systemic perspective within an organizational setting. However, they also foreground the importance of scientific evaluation of this method’s effectiveness. The perception of its effectiveness among the respondents combined with particular characteristics viewed as beneficial for organizational counseling offer important directions for future studies and thus the creation of evidence-based practices. In light of the broad and international scale at which the method is currently being applied, and the increased number of clients who are gaining exposure to this method, its scientific advancement appears to be critically important.

## Electronic Supplementary Material

Below is the link to the electronic supplementary material.

Supplementary Material 1

Supplementary Material 2

Supplementary Material 3

Supplementary Material 4

## Data Availability

The dataset are available for re-use upon request. Please contact the authors for access to the data.

## References

[CR1] Andrews M, Smits SJ (2018). Knowing what we know: uncovering Tacit Knowledge for Improved Organizational performance. J Organizational Psychol.

[CR2] Barton J, Emery M, Flood RL, Selsky JW, Wolstenholme E (2004). A maturing of systems thinking? Evidence from three perspectives. Systemic Pract Action Res.

[CR3] Bierema LL (2003). Issues involving practice-based learning and Improvement Systems thinking: a New Lens for old problems. J Contin Educ Health Prof.

[CR4] Boland H, Michaelis T (2006). Jenseits des Rubikon? Der mögliche Beitrag von Systemaufstellungen und Lösungsorientierung zur Beratungsarbeit in der Landwirtschaft [Beyond the Rubicon? The potential contribution of systemic constellations and solution orientation to extension work in agriculture]. Berichte über Landwirtschaft Zeitschrift für Agrarpolitik und Landwirtschaft.

[CR5] Burchardt C (2015) Business coaching and consulting - the systemic constellation approach in business. In: Schabacker M, Gericke K, Szélig N, Vajna S (eds) Modelling and management of engineering processes. Springer-Verlag, pp 101–112

[CR6] Checkland P (2000). Soft systems methodology: a thirty year retrospective. Syst Res Behav Sci.

[CR7] Fasching C (2009) Aufstellungen in Systemen. Unterschiede in Theorie und Anwendung von Aufstellungsmethoden aus der Erfahrungswelt österreichischer Aufsteller [Constellations in systems. Differences in theory and application of constellation methods from the experience of Austrian constellators], [Thesis, WU Vienna, Austria]. https://www.yumpu.com/de/document/read/6767140/diplomarbeit-osterreichisches-forum-systemaufstellungen

[CR8] Grant A, O’Connor S (2019). A brief primer for those new to coaching research and evidence-based practice. Coaching Psychol.

[CR9] Groth T (2004). Organisationsaufstellung — ein neues Zauberinstrument in der Beratung? [Organisational constellation - a new magic tool in consulting?]. Gruppendynamik und Organisationsberatung.

[CR10] Gutmark BJ (2014) Systemische Aufstellungen im organisationalen Kontext. Eine empirische Untersuchung zur Wirksamkeit von Organisationsaufstellungen [Systemic constellations in an organisational context. An empirical study of the effectiveness of organisational constellations], [Doctoral dissertation, Technical University Darmstadt, Germany]

[CR11] Harrington HJ, Carr JJ, Reis RP (1999). What’s this “systems” stuff. anyhow? The TQM Magazine.

[CR12] Hawkins P (2019) Systemic Team Coaching. In: Clutterbuck D, Gannon J, Hayes S, Iordanou I, Lowe K, MacKie D (eds) The practitioner’s handbook of Team Coaching. Routledge, pp 36–52

[CR13] Hwang A (2000). Toward fostering Systems Learning in Organizational Contexts. Systemic Pract Action Res.

[CR14] Kolodej C, Schröder J, Kallus KW (2016). Evaluation systemischer Strukturaufstellungen im Organisationskontext [Evaluation of systemic structural constellations within an organisational context]. Gruppe Interaktion Organisation Zeitschrift für Angewandte Organisationspsychologie (GIO).

[CR15] Konkolÿ Thege B, Petroll C, Rivas C, Scholtens S (2021). The effectiveness of Family Constellation Therapy in improving Mental Health: a systematic review. Fam Process.

[CR16] Kopp U, Martinuzzi A (2013). Teaching sustainability leaders in Systems thinking. Bus Syst Rev.

[CR17] Lam A (2000). Tacit knowledge, organisational learning and societal institutions: an integrated framework. Organ Stud.

[CR18] Lawrence P (2021). Team coaching: systemic perspectives and their Limitations. Philos Coaching.

[CR19] Maes G, Van Hootegem G (2019). A Systems Model of Organizational Change. J Organizational Change Manage.

[CR20] Scholtens S, Petroll C, Rivas C, Fleer J, Konkolÿ Thege B (2021). Systemic constellations applied in organisations: a systematic review. Gruppe Interaktion Organisation Zeitschrift Für Angewandte Organisationspsychologie (GIO).

[CR21] Schumacher T (2000) Systemische Strukturen in Familie und Organisation - Eine Studie zu Auswirkungen von Familienaufstellungen auf subjektive beziehungsbilder [Systemic structures in family and organisation - a study on the effects of family constellations on subjective relationships]. Verlag Rheintal Institute

[CR23] Segal ZV, Wiliams JM, Teasdale JD (2013) Mindfullness-based cognitive therapy for Depression, 2nd edn. The Guildford Press

[CR22] Senge PM (1990) The fifth discipline: the art and practice of the learning organization. Doubleday/Currency

[CR24] Sipman G, Thölke J, Martens R, Mckenney S (2022). Can a systemic-phenomenological teacher Professional Development Program Enhance awareness of Intuitions and serve pedagogical tact?. Systemic Pract Action Res.

[CR25] Tenner C (2014). Organisational Constellations in North America. Research Findings on Facilitators’ perspective. The Knowing Field.

[CR26] Wakefield K (2014) Coaching complexity: Exploring systemic coaching in practice. [Master thesis, University of Reading]

[CR27] Weinhold J, Bornhäuser A, Hunger C, Schweitzer J (2014) Dreierlei Wirksamkeit. Die Heidelberger Studie zu Systemaufstellungen [Triple efficacy. The Heidelberg study on system constellations]. Carl-Auer Verlag

