# Supplementary material for: A Systemic Perspective on Organizations: International Experience with the Systemic Constellation Method
Source: Syst Pract Action Res. 2023 Apr 10:1–18. Online ahead of print. doi: 10.1007/s11213-023-09642-2 (PMC10088654; doi:10.1007/s11213-023-09642-2)
Supplement: Supplementary file 1 — Supplementary Material 1 [file 11213_2023_9642_MOESM1_ESM.docx]

**A Systemic Perspective on Organizations:
International Experience with the Systemic Constellation Method**

**Online Supplementary Materials – 4**

**Tables**

#

**Table S1.** Responses to the open-ended question, “What sparked your interest in the method?”

| **Code** | | **Description** | **Examples of responses** | **N (%)*** |
| --- | --- | --- | --- | --- |
| ***Aspects relating to the characteristics of the method*** | | |  |  |
|  | **Perceived effectiveness** | Respondents noted that the method’s perceived effect and the insights acquired from using the method sparked their interest. They mentioned a long-lasting effect, a solution-focused orientation, help with decision making, and the ability of the method to loosen a system or let it 'flow' again. | *“The first constellation I was invited to was so powerful; my decision was made immediately right there and then.” “The power, speed, and depth of the method appeals to me.”* | 59 (22) |
|  | **Depth** | Respondents described the depth, richness, or wholeness that the method provides as sparking their interest. They mentioned that the method touches on the heart of an issue. | *“Its accuracy, depth, novelty, focus on groups/systems as a whole and its tremendous potential in organizations.” “Trust in the unknown; feeling connected with everything and everyone.’* | 28 (10) |
|  | **Embodiment** | Respondents mentioned embodiment or the use of sensations instead of cognition and verbal expressions as sparking their interest. It provides a “different language,” by allowing for communication without words. | *“The power of body and intuition in recognizing what is.”*  *“Making pre-verbal knowledge visible.”* | 28 (10) |
|  | **Visualization** | The ability to visualize a social system or a situation sparked the interest of the respondents. | *“To make visible.”  “Visual representation of the system.”* | 24 (9) |
|  | **Systemic perspective** | Respondents specifically mentioned the systemic perspective or the alternative view the method provided as an advantage | *“Systemic viewing, zooming out, the influence of dynamics on individuals and groups. Impact of history on behavior in the present and future.” “The system structure with the orders that are missing in therapy.”* | 22 (8) |
|  | **Healing** | Respondents mentioned that the “healing” effect of the method sparked their interest. | *“Learning about a powerful healing tool that deals with the root cause of issues.”* | 6 (2) |
| ***Aspects relating to coaches or consultants*** | | |  |  |
| **Professional opportunity** | | Respondents indicated that the potential they saw in the method for addressing professional problems, questions, or aspects of their professional lives sparked their interest. | *“…its tremendous potential in organizations.” “The possibility to expand the practical work and support clients.”* | 21 (8) |
| **Enriching toolbox** | | Respondents stated that the method was a valuable addition to their existing repertory that was relevant to their work. The innovative nature of the method sparked their interest, and they saw the method as an enriching tool. | *“I say this is a nice addition to the methods that I already use.”*  *“I wanted to broaden my expertise and incorporate a new method.”* | 15 (5) |
| **Personal or professional experience** | | Respondents encountered this method during a personal or professional experience, for instance, through a family constellation. They did not mention specifically what sparked their interest. | *“I encountered the method while I was on the path of my own personal development.”  “In my work as a consultant, I came across the method.”* | 98 (36) |
| **Recommendation** | | Respondents mentioned that the method was recommended by someone else, either as a specific name or a description, without reference to a personal or professional experience. They did not mention specifically what sparked their interest. | *“A friend recommended the trainer and I went to one of his one-day workshops and was hooked from then on.” “A good friend of mine introduced the systemic method to me.”* | 24 (9) |

Notes: *Out of 273 respondents, 230 (84%) responded to the question. Respondents could provide multiple responses, which may have resulted in multiple codes per respondent. A total of seven answers were unclear and could not be coded.

**Table S2.** Responses to the open-ended question, “What would be, for you, most helpful to improve the quality of your work?”

| **Code** | | **Description** | **Examples of responses** | **N (%)*** |
| --- | --- | --- | --- | --- |
| ***Needs relating to the characteristics of the method*** | | |  |  |
|  | **More research** | Respondents expressed a wish for more research and the establishment of a more fundamental scientific basis for the method. | *“More data on the efficacy of the method, even qualitative.”*  *“Scientific anchoring.”* | 18 (7) |
|  | **Materials** | Respondents mentioned that they would benefit from the availability of a good description of method or a manual, guidebook, or video. | *“A guide book/user manual with typical elements and structures and sentences.”*  *“A scientific explanation of how the method works; more extensive literature on the topic.”* | 18 (7) |
|  | **Certification** | Respondents mentioned that certification or improved quality or professionalism, possibly by means of more qualified training, would be helpful for them. | *“Concise shared agreements about key aspects of theory and practices...maybe clearer practitioner certification.”*  *“To offer the training at the university level including relevant theories, literature, assignments, workshops, etc. This is the main way of assuring the quality of the method and its development.”* | 8 (3) |
| ***Needs relating to the clients or the areas of application*** | | |  |  |
|  | **More acceptance** | Respondents indicated that it would be helpful for them if the method became more accepted, attained wider recognition, or became better known. | *“Greater recognition by the general public and corporate hierarchy that this is a useful and effective methodology.”*  *“Greater awareness of the method's legitimacy and efficacy within the market.”* | 20 (7) |
|  | **Broader application** | Respondents indicated that increased possibilities of applying the method or an increase in clients would be helpful. | *“More clients.”*  *“More occasions to do systemic work.”* | 7 (3) |
| ***Needs relating to coaches or consultants*** | | |  |  |
|  | **Peer-to-peer support** | Respondents referred to peer-to-peer support or feedback from peers as helpful for them to improve the quality of their work. | *“Regular exchanges with like-minded people, being able to experience each other’s work and get feedback.”*  *“Intervision groups and ongoing research, including references to professional resources and materials.”* | 34 (12) |
|  | **More practice** | Respondents desired more practice or experience with the application of the method, including adopting different roles and working in different fields and settings. This code referred both to their own practice and to the availability of opportunities to practice in general and receive feedback from clients. | *“More practice and feedback from clients over time.”*  *“Practicing and exploring plus supervision.”* | 32 (12) |
|  | **Continuous learning** | Respondents wished to increase their knowledge or continue their learning. They also desired more self-development in general. This code refers to their own learning and development and not to the availability of education in the field (see the code, Certification). | *“Continuous learning”*  *“Having more practice but also self-development by being the ‘client’ too.”* | 31 (11) |
|  | **Community and exchange** | Without specifically mentioning peer-to-peer support or supervision, the respondents expressed a desire to connect with other professionals. They wished to be part of a community or network or to participate in (international) exchanges. | *“To continue being a part of a network of facilitators.”*  *“Connecting back in with learning and the wider community.”* | 19 (7) |
|  | **Supervision** | Respondents mentioned that supervision or working with a more experienced professional would be helpful. | *“Community of practice and supervision.”*  *“To facilitate together with an experienced facilitator. Not in a course but in real life assignments.”* | 17 (6) |
|  | **Observe others** | Without specifically mentioning peer-to-peer support or supervision, respondents indicated that observing other professionals would be helpful. | *“To be able to see and do as many cases across different industries as possible.”*  *“More observation of different facilitators working with groups.”* | 8 (3) |
|  | |  |  |  |
| **Nothing** | |  |  | 6 (2) |
| **I don't know** | |  |  | 6 (2) |

Notes: *Out of 273 respondents, 157 (58%) responded to the question. Respondents could provide multiple responses, which may have resulted in multiple codes per respondent. A total of 30 answers were unclear and could not be coded.

**Table S3.** Responses to the open-ended question, “What does the field of the systemic organizational constellation method need?”

| **Code** | **Details** | **Examples of responses** | **N (%)*** |
| --- | --- | --- | --- |
| ***Needs related to the characteristics of the method*** | |  |  |
| **Research** | Respondents mentioned the need for a stronger scientific foundation or more studies on the method’s effectiveness. They also mentioned the need for a better understanding of the method. | *“Good research.”*  *“More scientific and rational thinking.”*  *“Documented case studies of ongoing work with organizations.”* | 27 (10) |
| **Materials and education** | Respondents mentioned that the field would benefit from the availability of a good description of the method or a manual or guidebook. Moreover, more high-quality education that is less costly or more accessible and more practice opportunities were felt needs. | *“Greater literature and evidence base.”*  *“Training programs to include [a section on] how to introduce the method to clients.”* | 18 (7) |
| **Less mystical** | Respondents disliked the mystical or esoteric impression that the method could convey; they felt that there should be less emphasis on “gurus” and more on the actual method. Moreover, they mentioned the need for more clarity regarding the method. | *“Professionalization (not esoteric practitioners who practice voodoo).”*  *“I am very shocked by the esoteric current within the field of system settings; it is much too mystifying.’* | 7 (3) |
| ***Needs relating to the clients or areas of application*** | |  |  |
| **Marketing** | Respondents indicated that the method should be promoted or marketed to a greater extent. | *“I think that more concrete successful stories should be shared.”*  *“Education of the public. Gentle/delicate promotion.”*  *“An awareness and marketing campaign.”* | 27 (10) |
| **More acceptance** | Respondents indicated that it would be helpful for them if the method became more accepted or gained greater recognition. | *“Greater communication, appreciation, and recognition by society.”*  *“Recognition by a wider public.”* | 21 (8) |
| ***Needs relating to coaches or consultants*** | |  |  |
| **Quality** | Respondents mentioned an improvement in the quality of the professionals who use the method, or more clarity regarding the quality of these professionals. They wished for further professional development within the field, possibly by means of certification or supervision. | *“Some kind of assessment of the competencies and code of ethics.”*  *“Mature, reasonable, and highly qualified professional people, teams, and organization developers, who incorporate this method in their portfolios.”*  *“Clarity about qualifications, certification, awarding bodies, and supervision.”* | 24 (9) |
| **Unity** | Respondents disliked the competition between different professionals, different schools, or between this and other methods. They sought more unity or openness. | *“Less competition between different providers.”*  *“Unity consciousness....I have witnessed divisiveness and competition among practitioners and facilitators.”* | 19 (7) |
| **Community** | Respondents wished for an organized (international) community. | *“More international collaboration and sponsorship.”*  *“A community to share best practices and failures.”* | 11 (4) |
| **Self-confidence** | Respondents mentioned that the method should mature and that professionals working with the method show more self-confidence. | *“Self-confidence.”*  *“Mature and take more space.”*  *“The field demands experience, trust, and autonomy.”* | 10 (4) |
|  |  |  |  |
| **Nothing** |  |  | 3 (1) |
| **I don't know** |  |  | 12 (4) |

Notes: *Out of 273 respondents, 160 (59%) responded the question. Respondents could provide multiple responses, which may have resulted in multiple codes per respondent. A total of 37 responses were unclear and could not be coded.
