# Supplementary material for: A Systemic Perspective on Organizations: International Experience with the Systemic Constellation Method
Source: Syst Pract Action Res. 2023 Apr 10:1–18. Online ahead of print. doi: 10.1007/s11213-023-09642-2 (PMC10088654; doi:10.1007/s11213-023-09642-2)
Supplement: Supplementary file 2 — Supplementary Material 2 [file 11213_2023_9642_MOESM2_ESM.docx]

**A Systemic Perspective on Organizations:
International Experience with the Systemic Constellation Method**

**Online Supplementary Materials – 2**

**Survey**

**Welcome to the questionnaire on the application and use of the systemic constellation method in organisations.**

**Aim of the research**

The international field of consultants, coaches, trainers, and other professionals who use the systemic constellation method in organisations is diverse and has grown enormously in the last decade. The aim of this study is to evaluate the use of the systemic constellation method in organisations from the perspective of professionals who use the method. As an additional aim, we are also interested in gathering a more comprehensive understanding of their perspectives on the method in general.

**Procedure**

We would like to ask you to fill out an online questionnaire, which will take approximately 20 minutes and consists of questions about your personal experience with the method and how you apply it in your work. Additionally, we ask about your opinion and point of view regarding the method. The questionnaire is available in five different languages (English, Dutch, Spanish, Portuguese, and German) and can be completed anonymously. We would also greatly appreciate it if you would forward the questionnaire to anyone you know who might be interested.

**Research results**

This questionnaire is administered to the international community of consultants, coaches, trainers, and other professionals who use the systemic constellation method in organisations. The questionnaire is distributed via Infosyon (the International Forum for System Constellations in Organisation, www.infosyon.com), a number of large training institutes, and other relevant networks. We will use your responses to evaluate the perspectives, opinions, and needs of people who use the method. This will contribute to a better understanding of the method and further improve quality of training on an international level. We will share our insights as soon as possible via the channels mentioned above. If you are interested in receiving the results of the study directly, you are welcome to provide your email address at the end of the questionnaire.

**Confidentiality**

The results of this study will be treated confidentially and anonymously. Your data will be processed by means of a participant number and stored within the IT systems of the University Medical Center of Groningen (UMCG), the Netherlands. The data will only be used for research regarding the systemic constellation method. There are no foreseeable risks involved.

Please note that if you close the questionnaire before finishing it, your answers will be saved for 2 weeks on that device. You have *2 weeks* to finish the questionnaire on the same device. After that period, your answers will no longer be available on that device.

**Further information**

If you have questions about this study, you may ask before or after completing the questionnaire. Please contact the investigator who is responsible for this study

Thank you in advance for your support!

*I hereby consent to have read and understood the above elaborated study conditions:*

- Yes I consent
- No I do not consent

🡪 You have decided not to take part in our study. In the next screen the survey will close. We want to thank you for your time and interest regardless!

In the box below, you may comment on your reasons not to consent, but of course, you can also leave this box empty.

**Before we start the questionnaire, we would like to ask you a few questions about yourself**

1. Which option most accurately describes your current working situation (in general)?

- Self-employed / Business owner with no personnel
- Self-employed / Business owner with personnel
- Employed - fixed contract
- Employed - temporary contract
- Unemployed or unable to work
- Retired
- Other, namely <open question>
- I prefer not to say

2. What is your native language?

< choose >

- English
- German
- Spanish
- French
- Portuguese
- Chinese (Mandarin)
- Dutch
- Danish
- Other, namely <open question>
- I prefer not to say

3. What is your nationality?

<open question>

4. In which country do you generally work most often with the systemic organisational constellation method?

< choose from Qualtrics format >

5. What gender do you identify with?

- Male
- Female
- Other or I prefer not to say

6. What is your age?

- 25 years or younger
- 26- 40 years
- 41 - 55 years
- 56 - 70 years
- 71 years or above
- I prefer not to say

**The following questions refer to the systemic constellation method applied in organisations or for societal issues (in short: systemic organisational constellation method). This can be any kind of organisation or issue, as long as it does not have its primary focus on clinical or family counselling. With the systemic organisational constellation method, we mean a systemic perspective in combination with constellations with people or with objects (for instance table-top-constellations) or constellations in mind. You do not have to do a constellation every time you use the method.**

**Also, we are well aware that there are many different variants of the systemic organisational constellation methods and schools, but for the purpose of this study, we do not differentiate and instead we refer to ‘the’ systemic organisational constellation method.**

7. Have you ever used the systemic organisational constellation method?

- Yes
- No
- I don’t know

8. Do you plan to use the systemic organisational constellation method in the future?

- Yes
- No 🡪 questionnaire ends, see popup
- I don’t know 🡪 questionnaire ends, see popup

Pop up:

Dear participant, we are grateful for your support. However, this questionnaire is specifically for people who use the systemic organisational constellation method or are planning to use it. Since you replied “no” or “I don’t know” on both of these questions, we will end the questionnaire here.

🡪 End of questionnaire and forward to interest mail

9. In what year did you begin using the systemic organisational constellation method?

- Before 1990
- Between 1990 and 1995
- Between 1996 and 2000
- Between 2001 and 2005
- Between 2006 and 2010
- Between 2011 and 2015
- After 2015
- I don’t remember

10. Do you still use this method (now and then)?

- Yes
- No
- I don’t know or I prefer not to say

11. Why don’t you use the method anymore?

- <open question>

12. How often do you use this method?

- once per week or more
- once per month or more, but less than once per week
- once per year or more, but less than once per month
- I don’t know or I prefer not to say

13. Which answer best describes your role when using the systemic organisational constellation method? If multiple options apply, please choose the function that fits best.

- Facilitator
- Constellator
- Trainer
- Coach
- Consultant / advisor
- (Project)manager
- HR manager or HR specialist
- CEO, business owner
- Teacher, docent
- Researcher
- Therapist
- Other

🡪 Please specify <open question>

- I don’t know or I prefer not to say

14. How many years of (part-time) education or training do you have, on estimation, regarding the systemic organisational constellations method?

- < Number > (1 t/m 50)
- I don’t know
- No training at all

15. Where or from whom did you receive your education or training in the systemic organisational constellation method?

- I got my education or training from… <open question>
- I don’t know or I prefer not to say

16. What sparked your interest in the method?

- This sparked my interest… <open question>
- I don’t know or I prefer not to say

17. What are the personal advantages that you experience from using the method? This can include direct and monetary advantages, but also indirect advantages on, for instance, a personal level.

- The advantages are… <open question>
- I don’t know or I prefer not to say

18. What are the personal disadvantages that you experience from using the method? This can include direct and monetary disadvantages, but also indirect disadvantages on, for instance, a personal level.

- The disadvantages are… <open question>
- I don’t know or I prefer not to say

**The following questions refer to how you use the method**

19. When using the systemic organisational constellations method, how often do you perform a constellation with human representatives?

- Never
- Rarely
- Sometimes
- Regularly
- Often
- Always
- I don’t know or I prefer not to say

20. How often do you combine the systemic organisational constellations with other methods?

- Never
- Rarely
- Sometimes
- Regularly
- Often
- Always
- I don’t know or I prefer not to say

21. With which therapy form(s), school(s), or method(s) do you sometimes combine the systemic organisational constellations method? You can choose multiple methods.

- Cognitive behavioral therapy
- Gestalt
- Depth Psychology
- Large Group Interventions
- Group or team dynamics
- Team coaching
- Theory U
- Deep democracy
- General organisational development techniques
- Soft Systems Methodology
- Psychodrama / Sociodrama
- Drama
- Systemic Therapy excluding constellations
- Other 🡪 < Open question>
- I don’t know or I prefer not to say

22. In which setting(s) do you apply systemic organisational constellations? You can choose multiple settings.

- Individual coaching
- Team coaching
- Organisational coaching or consultancy
- Education
- Therapy
- Research
- Other - open question
- I don’t know or I prefer not to say

23. For which topic(s) or issue(s) do you apply systemic organisational constellations? You can choose multiple topics.

- Conflicts
- Team functioning
- Development of new insights
- Exploring new points of view
- Reorganisation
- Acquisition or takeover
- Succession
- Mediation
- Organisational development
- Organisational transformation
- Leadership development
- Strategy development
- Stakeholder analysis
- Research
- Other <open question>
- I don’t know or I prefer not to say

24. In which industries, sectors or type of organisations have you already done systemic organisational constellations? For instance, agriculture, finance, primary education, retail, transportation, etc.

- <open question>

25. How big were the companies or institutes in which you did systemic organisational constellations in terms of employees?

- <open question>

26. In your opinion, do you have to be familiar with the intrinsic laws and values of a company or industry to be able to make effective systemic organisational constellations?

- Yes
- No
- To some extent
- I don’t know or I prefer not to say

27. In the past three years, have you encountered a situation in which the method was applied in a surprisingly successful or unsuccessful way? This may include positive or negative experiences.

- Yes
- No
- I don’t know or I prefer not to say

28. Could you please elaborate a bit on this experience?

- <open question>

29. Have you ever felt that the methods might have had a counterproductive effect on the organisation and/or the person(s) you worked with?

- Yes
- No
- I don’t know or I prefer not to say

30. Could you please elaborate on this experience? What might have been a cause and how could it have been prevented?
<open question>

31. Do you have experiences with online constellations in your role as facilitator, trainer or coach?

- Yes
- No
- I don’t know or I prefer not to say

32. How did you experience online constellations in the function as facilitator?

- < Open question >

33. Did the experience with online constellations change your view on the method?

- Yes
- No
- I don’t know or I prefer not to say

34. Could you please elaborate on this experience?

- <open question>

**The following questions refer to your needs or wishes as a user of the method**

46. What would be, for you as a user of the method, most helpful to improve the quality of your work?

- < Open question >

47. What does the field of systemic organisational constellation method need?

- < Open question >

48. Do you have any remaining thoughts about the method that you want to share?

- < open question >

49. Are you a member of Infosyon (the International Forum for System Constellations in Organisation, www.infosyon.com)?

- Yes
- No
- I don’t know or prefer not to say

50. Are you a member of another relevant network?

- Yes, namely < open question >
- No
- I don’t know or prefer not to say

Split up questionnaire here. People are redirected to a new questionnaire. Email will not be saved together with answers.

**We have come to the end of the questionnaire. If you are interested in the outcomes of this research or other research activities, please leave your email address below. This email address will be stored separately from the answers on the questionnaire.**

- email address

**Based on the data from this questionnaire, we might want to pursue further research. Would you be available for additional questions? If so, please leave your email address below. You can still decide on your participation when we contact you. This email address will be stored separately from the answers on the questionnaire.**

- Yes 🡪 email address
- No

Thank you for your participation and support. This will help us to evaluate the use of the systemic organisational constellation method in organisations from the perspective of professionals who use the method.
