# Supplementary material for: A Systemic Perspective on Organizations: International Experience with the Systemic Constellation Method
Source: Syst Pract Action Res. 2023 Apr 10:1–18. Online ahead of print. doi: 10.1007/s11213-023-09642-2 (PMC10088654; doi:10.1007/s11213-023-09642-2)
Supplement: Supplementary file 3 — Supplementary Material 3 [file 11213_2023_9642_MOESM3_ESM.docx]

**A Systemic Perspective on Organizations:
International Experience with the Systemic Constellation Method**

**Online Supplementary Materials – 3**

**Industries and sectors**

**Question:**

In which industries, sectors or type of organisations have you already done systemic organisational constellations? For instance, agriculture, finance, primary education, retail, transportation, etc.

**Collected answers:**

Administration services

Advertising

Aerospace

Agriculture

Architecture

Aviation, airlines

Bildung, personal development

Bio-technology

Car industry

Charity

Chemical industry

Churches and religious/spiritual organizations

Coaching

Community development

Construction

Consultancy

Consumer packages

Cultural sector (museum, theater)

Daycare centers

Dentistry

Education

Energy services

Fashion and clothing industry

Financial and professional services

Food industry

Government and municipalities

Health and medical services

Insurance

International development, diplomacy

International peace organisation

IT and telecommunication services

Logistics

Manufacturing

Marketing

Mechanical and electrical engineering

Media

Non-profit

Oil and gas production

Pharma industry

Public service

Real-estate

Recruitment

Research

Restaurants, cafe, hotels

Retail

Service sector

Social care

Sports

Sustainability

Trade, commerce

Transportation

Utilities (electricity, water, gas)

Veterinary
