# Supplementary material for: A Systemic Perspective on Organizations: International Experience with the Systemic Constellation Method
Source: Syst Pract Action Res. 2023 Apr 10:1–18. Online ahead of print. doi: 10.1007/s11213-023-09642-2 (PMC10088654; doi:10.1007/s11213-023-09642-2)
Supplement: Supplementary file 4 — Supplementary Material 4 [file 11213_2023_9642_MOESM4_ESM.docx]

**A Systemic Perspective on Organizations:
International Experience with the Systemic Constellation Method**

**Online Supplementary Materials – 1**

**Outline for the implementation of the Systemic Constellation method**

Salome Scholtens, Groningen University, the Netherlands

Refer to: Scholtens, S., Petroll, C., Rivas, C., Fleer, J., and Konkolÿ Thege, B. (2021). Systemic constellations applied in organisations: a systematic review. *Gruppe.Interaktion.Organisation. Zeitschrift Für Angewandte Organisationspsychologie (GIO)*, *52*, 537–550.

In general, the procedure of the Systemic Constellation method follows a number of steps described below. At the start, the consultant welcomes the group, and introduces the session and provides information on practical aspects. Once either the consultant, a participant or the group has decided on who will participate in the interview, the consultant interviews a participant (the focal person) regarding a team or organizational issue. Together they try to gain a clearer view on the issues or unravel the starting question. The idea is that this starting question is the entry into an underlying, often implicit mental image of a certain situation or issue in a social system. The knowledge people have on a social system often entails implicit, tacit knowledge that is difficult to verbalize and make explicit. For a constellation, the issue or question does not need to be very clear, just clear enough to be able to select elements that are relevant in this case.

In the next step, the consultant and the focal person jointly identify elements that are relevant for the given case and create a visualization of the social system using these elements (the constellation). The other participants who are present during the constellation also actively participate as representatives or as observers. These representatives do not require any knowledge on the issue that is addressed. Also, they do not play a part or get instructions, like in regular role play methods.


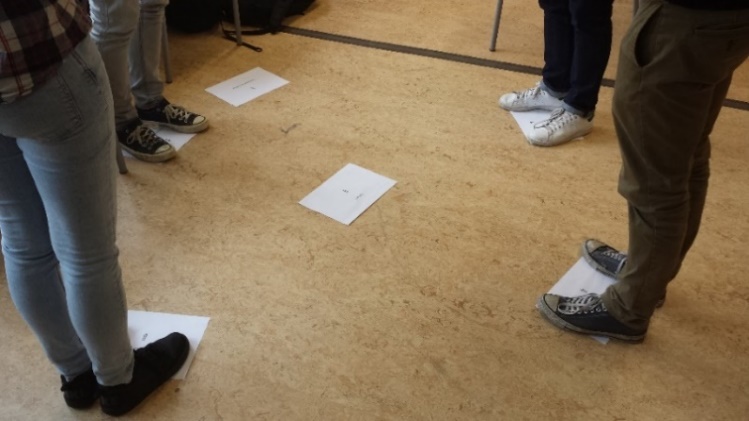
The consultant examines the constellation
together with the focal person and invites the
focal person to literally try different perspectives. Based on the image that is emerging, additional questions can be asked by the consultant from
the focal person, the representatives or the observers, which can lead to the addition,
removal or rearrangement of an element in
the constellation. This way, the constellation session may go through a number of successive rounds until a point of saturation.

Finally, the constellation is closed. The consultant helps the client to incorporate the experience and the new image. The session closes with a short debriefing where experiences and insights can be shared.
